# Evidence synthesis - COVID-19 among Black people in Canada: a scoping review

**DOI:** 10.24095/hpcdp.44.3.05

**Published:** 2024-03

**Authors:** Adedoyin Olanlesi-Aliu, Janet Kemei, Dominic Alaazi, Modupe Tunde-Byass, Andre Renzaho, Ato Sekyi-Out, Delores V. Mullings, Kannin Osei-Tutu, Bukola Salami

**Affiliations:** 1 Faculty of Nursing, University of Alberta, Edmonton, Alberta, Canada; 2 Health and Immigration Policies and Practices Research Program (HIPP), University of Alberta, Edmonton, Alberta, Canada; 3 Black Physicians of Ontario, Toronto, Ontario, Canada; 4 Department of Obstetrics and Gynecology, University of Ontario, Toronto, Ontario, Canada; 5 Translational Health Research Institute, School of Medicine, Campbell Town Campus, Western Sydney University, Australia; 6 Black Opportunity Fund, Toronto, Ontario, Canada; 7 School of Social Work, Memorial University, St John’s, Newfoundland and Labrador, Canada; 8 Department of Family Medicine, University of Calgary, Calgary, Alberta, Canada; 9 Department of Community Health Sciences, University of Calgary, Calgary, Alberta, Canada

**Keywords:** racialized populations, inequity, vaccine hesitancy, racial discrimination

## Abstract

**Introduction:**

The COVID-19 pandemic exacerbated health inequities worldwide. Research conducted in Canada shows that Black populations were disproportionately exposed to COVID-19 and more likely than other ethnoracial groups to be infected and hospitalized. This scoping review sought to map out the nature and extent of current research on COVID-19 among Black people in Canada.

**Methods:**

Following a five-stage methodological framework for conducting scoping reviews, studies exploring the effects of the COVID-19 pandemic on Black people in Canada, published up to May 2023, were retrieved through a systematic search of seven databases. Of 457 identified records, 124 duplicates and 279 additional records were excluded after title and abstract screening.Of the remaining 54 articles, 39 were excluded after full-text screening; 2 articles were manually picked from the reference lists of the included articles. In total, 17 articles were included in this review.

**Results:**

Our review found higher rates of COVID-19 infections and lower rates of COVID-19 screening and vaccine uptake among Black Canadians due to pre-COVID-19 experiences of institutional and structural racism, health inequities and a mistrust of health care professionals that further impeded access to health care. Misinformation about COVID-19 exacerbated mental health issues among Black Canadians.

**Conclusion:**

Our findings suggest the need to address social inequities experienced by Black Canadians, particularly those related to unequal access to employment and health care. Collecting race-based data on COVID-19 could inform policy formulation to address racial discrimination in access to health care, quality housing and employment, resolve inequities and improve the health and well-being of Black people in Canada.

**Highlights**

- Black Canadians are overrepresented in frontline jobs, which increases their risk of contracting COVID-19.
- Low uptake of COVID-19 screening and vaccine hesitancy may be attributed to mistrust of the health care system in Canada.
- Existing structural racism within the Canadian health care system has created inequities in accessing COVID-19–related health care services among Black Canadians.
- There is a need to collect racebased data with a focus on resolving inequities and improving the health and well-being of Black people in Canada.

## Introduction

The World Health Organization declared COVID-19 a global pandemic in March 2020, triggering the adoption of numerous public health measures, including lockdowns, social distancing and the use of facemasks in public places. However, the health risks of COVID-19 infection and public health measures to reduce infection did not affect everyone equally;^1^-^5^ the burden was disproportionately greater for racialized people and those living in low-income communities.^6^

Black people in Canada, the United Kingdom (UK) and the United States (USA) experienced a disproportionately higher prevalence of COVID-19 infections and a greater risk of COVID-19-related hospitalizations and mortality compared to their White counterparts.^7^-^13^ For every 100000 Americans, about 26 Black people died from COVID-19 infection, a mortality rate more than twice that of Latino, Asian or White people.^13^ In the UK, the mortality rate among Black people was likely four times that of their White counterparts.^14^-^17^ Despite representing only 9.28% of the population of Toronto, Ontario, Canada’s largest city, Black people accounted for nearly one-quarter of COVID-19 cases in 2020, while White people, who constituted 49.64% of the city’s population, represented only 21.7% of cases.^18^

Canada is a common destination for international migrants, with a growing population of Black people from sub-Saharan African and Caribbean nations.^19^ Black people are the third-largest racialized group in Canada, at 4.3% of the country’s total population, after South Asian (7.1%) and Chinese (4.7%) people.^20^

Most of the reasons why the Black population was highly susceptible to and affected by COVID-19 infection are rooted in social determinants of health, such as socioeconomic status, crowded living environments, cultural barriers, racial discrimination, poor access to health care and anti-Black racism.^21^ In Canada and the USA, systemic racism cuts across all sectors—health care, education and the labour force—a problem that continues to be overlooked in policies.^21^,^22^ Because of the extensive emphasis on individual behaviours, rather than tackling the challenges that confront systemically marginalized Black people,^21^ the health care system failed to account for numerous inequities, including in education and employment, that tended to expose Black people to high rates of COVID-19 infection and mortality, to the point that racism has been described as “a risk factor for dying from COVID-19.”^23^

For instance, racialized and immigrant populations experienced unequal access to vaccination and high rates of infection and death from COVID-19.^24^,^25^ A significant proportion of Black people are precariously employed and overrepresented in risky but essential frontline jobs across Canada, as well as in the UK and the USA,^17^,^26^ where the risks of COVID-19 infection were high.^27^-^30^ Racial inequalities to do with health and environmental factors affect racialized people in a way that left them “more exposed [to] and less protected” from the COVID-19 virus.^8^,^23^,^30^

Data from the USA demonstrate racial disparities in rates of COVID-19 infection and mortality, with Black people among the most disadvantaged.^8^,^23^ However, few studies have focussed on COVID-19 among Black Canadians. Given the disproportionate burden of COVID-19 and the distinct risks that Black Canadians face, the purpose of this scoping review was to map out the scope of research on COVID-19 among Black people in Canada.

## Methodology

We utilized a scoping review methodology to explore the “extent, range and nature of research activity,”^31^ explicate what is currently known about COVID-19 among Black Canadians and pinpoint knowledge gaps for future research. We applied Arksey and O’Malley’s^31^ five-stage methodological framework for conducting scoping reviews: identifying the research question; identifying relevant studies; selecting studies; charting the data; and collating, summarizing and reporting the results. We used the Tricco et al.^32^ Preferred Reporting Items for Systematic Reviews and Meta-Analyses extension for Scoping Reviews (PRISMA-ScR) approach. 

***Identifying the research question***

This review was guided bythe following question: “What is the scope and nature of the literature on COVID-19 among Black people in Canada?” 

***Identifying relevant studies***

We identified relevant studies through a systematic search in seven electronic databases: Ovid MEDLINE (Table 1), Elsevier Embase, APA PsycINFO, CABI Global Health, EBSCO CINAHL, Elsevier Scopus and the Wiley Cochrane Library. Our search strategy was derived based on two main concepts: (1) COVID-19 (all variants), and (2) Black people in Canada.

**Table 1:** Search strategy

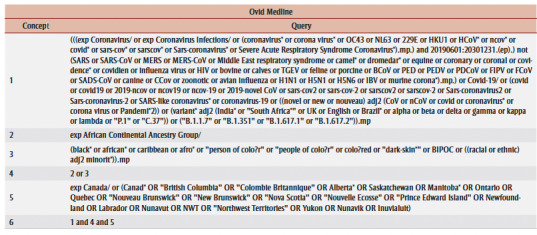

***Selecting studies***

Our initial search was conducted in January 2022 with no time restrictions. We subsequently updated the search to include all records published to 31 May 2023. A total of 457 records underwent initial screening of titles and abstracts (by AO and JK), and 124 duplicates were excluded.Two authors (AO and JK), working independently, reviewed the abstracts of the remaining 333 articles. Any conflicts during the process of selecting articles were resolved by a third author (DA). An additional 279 articles were excluded as they did not meet the inclusion criteria (studies focussing on Black people living in Canada and on COVID-19). Of the remaining 54 articles, 39 were excluded after the full-text screening. In total, 17articles were included in this review. 

**Figure 1:**
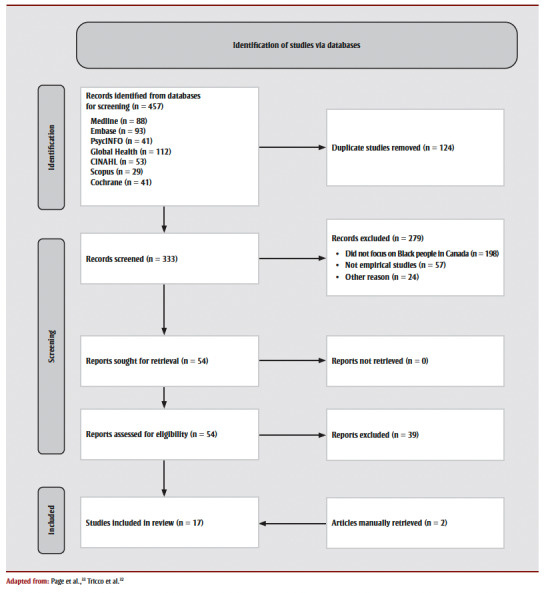
PRISMA 2020 flow diagram depicting identification of studies via databases on COVID-19 among Black people in Canada

The selection process is depicted in Figure1.

***Charting the data***

Two research team members (AO and JK) conducted the data extraction, which involved charting and sorting the findings of the included studies into key issues and analytical categories related to the impact of COVID-19 on Black people in Canada. The following information was extracted from each of the included articles and recorded on an Excel spreadsheet (version 2007; Microsoft Corp., Redmond, WA, US) designed by the research team: author name(s), year of publication, study purpose/research question, study population, methods, results/findings and comments/implications (Table 2). A research team member (DA) performed a quality check to ensure completeness and accuracy.

**Table 2:** Data extraction sheet

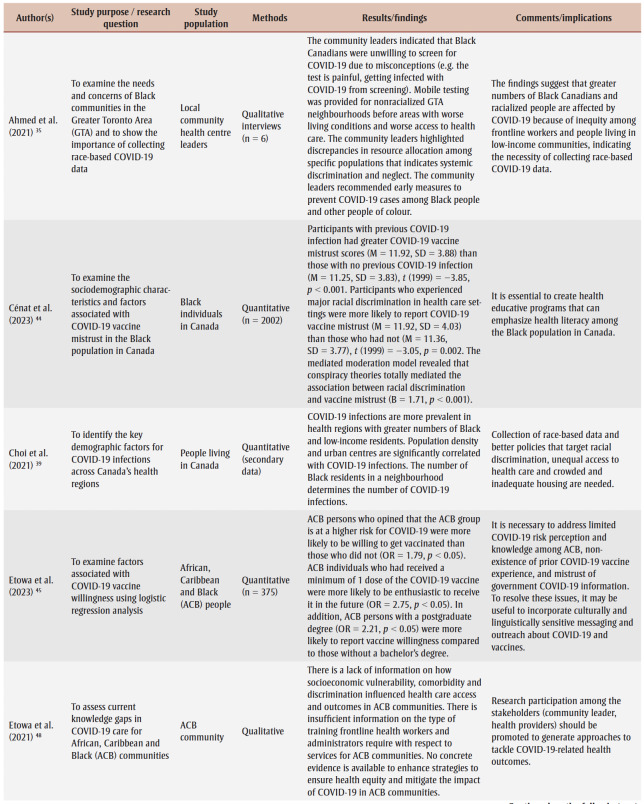 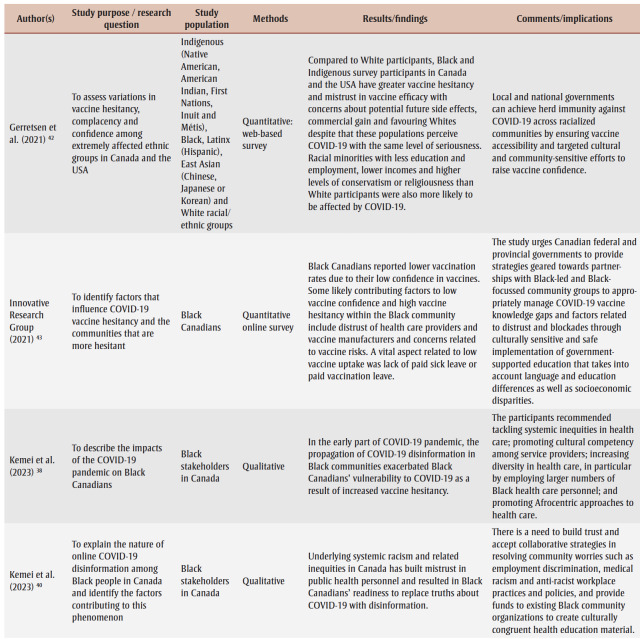 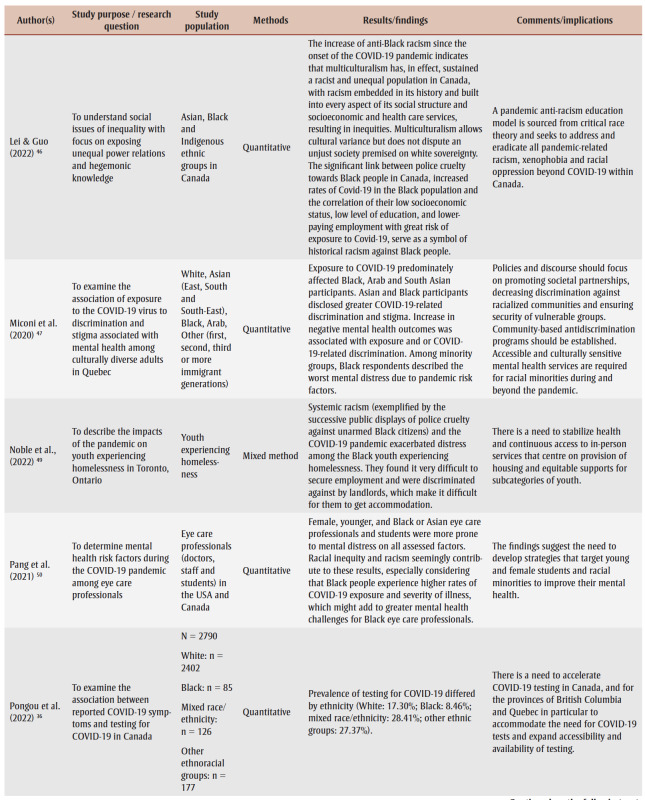 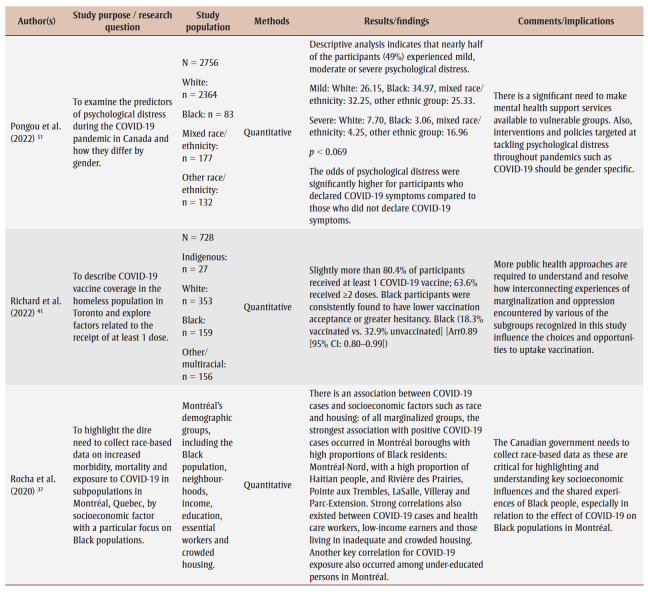

***Collating, summarizing and reporting the results***

We analyzed quantitative data from numerical summary and qualitative studies using thematic analysis. Drawing on Braun and Clarke,^34^ two research team members (AO and JK) read the included articles several times, familiarized themselves with the data, and synthesized and categorized the interpretations of recurring findings into themes. They then open coded the extracted data by going through the fragments of texts, line by line, and assigning labels that best described these fragments. The codes were then compiled into potential themes, all the data relevant to each potential theme were grouped together, and the data were compared across the coded excerpts and the entire dataset. Two other research team members (DA and BS) reviewed the assigned codes and themes.

***Ethics approval***

This scoping review does not contain any studies with human participants or animals that may have required ethics approval.

## Results

A total of 17 empirical studies met our inclusion criteria. Twelve articles used quantitative methodologies (mostly cross-sectional study designs), four used qualitative methodologies (mostly explorative) and one used mixed methods. All included articles described the impacts on Black Canadians of, for example, poor accessibility to COVID-19–related health care services, health inequities caused by COVID-19 and the role of systemic discrimination and racism in the creation of these inequities. 

Our findings are presented in five themes: low uptake of COVID-19 screening; high rates of COVID-19 infection; low uptake of COVID-19 vaccines; systemic racism and discrimination; and mental health impacts.

***Low uptake of COVID-19 screening***

Two studies reported on disparities in COVID-19 screening.^35^,^36^ In a cross-sectional study, Pongou et al.^36^ found that the prevalence of being tested for COVID-19 across reported COVID-19 symptoms was far lower among Black Canadians (8.46%) than among those who were White (17.30%), mixed race/ethnicity (28.41%) or from another ethnoracial group (27.37%), although the differences were not statistically significant.

In a 2021 qualitative study, local community health centre leaders who serve communities with large populations of racialized people within the Greater Toronto Area expressed concerns that individuals’ reluctance to get tested for COVID-19 were due to misconceptions that the test is painful and that people can get infected with COVID-19 from screening.^35^ The study also noted discrepancies in resource allocation within the health care system. For instance, mobile testing was made available in nonracialized neighbourhoods sooner than in poorer and racialized areas with worse access to health care.^35^

***High rates of COVID-19 infection among Black Canadians***

Four studies examined high rates of COVID-19 infection among Black Canadians.^36^-^39^ A quantitative study indicated that higher numbers of COVID-19 cases are associated with socioeconomic factors such as race and housing. For instance, of all marginalized groups in Montral, Quebec, the strongest relationship with positive COVID-19 cases occurred among those living in overcrowded housing and in boroughs with high proportions of Black people.^37^

Two qualitative studies reported on the greater risks for Black people of contracting COVID-19 as a result of overrepresentation in frontline work and low-income communities.^37^,^38^ Using COVID-19 counts and tabular census data, Choi et al.^39^ showed that there were relatively higher numbers of infections in communities with larger proportions of Black and low-income residents across Canada. These vulnerabilities were created by poverty, overcrowded living environments, predominance of frontline work and existing health care inequities.^36^,^37^

***Low uptake of COVID-19 vaccines***

Seven studies explored low uptake of COVID-19 vaccines among Black Canadians.^38^,^40^-^45^ A quantitative study exploring COVID-19 vaccine coverage among people experiencing homelessness in Toronto found that about 80.4% of participants received at least one dose of a COVID-19 vaccine and 63% had received two or more doses; however, Black participants were consistently found to have greater vaccine hesitancy, likely because of distrust in health care providers, perceived commercial gains for vaccine manufacturers, perception of vaccine risks, and lack of paid sick or vaccination leave.^41^

Two qualitative studies reported that the spread of disinformation and misinformation about COVID-19 within Black communities in Canada during the early part of the pandemic affected people’s understanding of the risk of the consequences of COVID-19 infection, which propagated vaccine hesitancy.^38^,^40^ Gerretsen et al.^42^ conducted a quantitative web-based survey in Canada and the USA to assess variations in vaccine hesitancy; the authors reported that despite perceiving COVID-19 with the same level of seriousness, Black and Indigenous people possess a greater degree of vaccine hesitancy and mistrust in efficacy than do White people due to concerns about potential future side effects and commercial gains and because they favoured natural immunity to a greater degree. 

Two quantitative study findings reported lower vaccination rates and higher vaccine mistrust scores among Black individuals as a result of their experiences of major racial discrimination in the health care system.^43^,^44^ In addition, a quantitative study of vaccine willingness found that African, Caribbean and Black individuals at greater risk of infection with COVID-19 were more willing to get vaccinated, and those who had received their first dose of the COVID-19 vaccine were more willing to receive upcoming doses.^45^

***Systemic racism and discrimination***

The five studies that explored the difficulties Black Canadians faced during the pandemic as a result of systemic racism and discrimination determined that the difficulties study participants experienced accessing COVID-19-related health care might have been exacerbated by existing stress stemming from racism, systematic bias and barriers, and socioeconomic vulnerabilities.^40^,^46^-^49^ The significant link between police cruelty towards Black people in Canada, increased rates of COVID-19 in the Black population and the correlation of lower socioeconomic status, lower level of education and lower-paying employment with great risk of exposure to COVID-19, serves as a symbol of the continuing and historical racism experienced by Black people.^46^

The underlying systemic racism and related inequities resulted in mistrust of health care providers.^40^ Miconi et al.^47^ conducted a mixed study on risk of exposure to COVID-19 and the relation to discrimination and stigma associated with mental health findings. The authors reported that Black, Arab and South Asian participants had higher prevalence of infection, while Black and Asian participants disclosing greater COVID-19-related discrimination and stigma as a result of their employment, for example, as frontline workers.^47^

The quantitative study conducted by Noble et al.^49^ revealed that systemic racism and the COVID-19 pandemic exacerbated mental distress among Black youth experiencing homelessness in Toronto, with barriers to securing employment and landlords’ racial discrimination making it difficult to obtain accommodation.

A qualitative study revealed that there was insufficient information on the type of training frontline health workers and administrators require when providing services to African, Caribbean and Black communities.^48^ No concrete evidence is available to enhance strategies to ensure health equity and mitigate the impact of COVID-19 in African, Caribbean and Black communities. 

***Mental health impacts***

Four studies reported on mental health impacts of the COVID-19 pandemic on Black people in Canada.^38^,^47^,^50^,^51^ A qualitative study found that online misinformation about COVID-19 aggravated mental health issues among Black Canadians and resulted in fear of and anger about mandatory vaccine orders.^38^ Some Black community members were afraid of being stigmatized whether they received or declined the COVID-19 vaccine, which amplified their anxiety about COVID-19 and prevented them from getting vaccinated or promoting vaccination.^38^

In their quantitative study on eye care professionals’ mental health risk factors during the COVID-19 pandemic, Pang et al.^50^ reported poor emotional health among Black and Asian optometrists, noting that they were more prone to mental distress and elevated symptoms of depression and anxiety than other ethnoracial groups. Miconi et al.^47^ reported an increase in negative mental health outcomes associated with exposure to the COVID-19 virus and/or COVID-19-related discrimination, with Black survey respondents describing the worst mental distress due to the pandemic. Further, research on predictors of psychological distress during the COVID-19 pandemic in Canada show that nearly half of the participants (49%) experienced mild, moderate or severe psychological distress, with Black Canadians (mild: 34.97%; severe: 3.06%) reporting a higher percentage of mild and severe psychological distress compared to White (mild: 26.15%; severe: 7.70%) and mixed race/ethnicity (mild: 32.25%; severe: 4.25%) Canadians.^51^

## Discussion

This scoping review identified five themes addressing the impact of COVID-19 on Black Canadians. Key among our findings is evidence of the inequalities in access to COVID-19–related health care, which may be attributed to existing structural racism within the Canadian health care system. Given the highly infectious nature of the disease, inequalities in access to care in the context of COVID-19 affects the entire population. For instance, anyone who does not adopt COVID-19 preventive measures, prompt diagnosis and treatment, may contract the virus and spread it in their community.

The COVID-19 pandemic accentuated health inequities based on anti-Black racism. Two studies in this scoping review showed that early measures to control the spread of COVID-19 (e.g. screening) were not effectively implemented in the areas where most Black people resided, which increased residents’ risk of infection.^35^,^36^ The COVID-19 mortality rate among Black people living in low-income areas was 3.5 times higher than in nonracialized and non-Indigenous populations living in low-income areas.^52^ Further, Black people have been at greater risk of hospitalization for and dying from COVID-19 due to inadequate access to health care providers and services.^53^,^54^ Other factors that contribute to the high rates of COVID-19 infection among Black people include poverty, poor and overcrowded living conditions, and employment in precarious frontline work.^55^,^56^ Communities in Canada with larger proportions of Black and racialized populations had higher rates of COVID-19 infection and death.^55^,^56^ For instance, Ontario is home to more than 50% of Canada’s Black population, and overrepresentation of COVID-19 cases were reported in Black neighbourhoods.^18^,^55^-^57^ Other studies conducted in Edmonton, Alberta,^58^ and Montral, Quebec,^59^ also revealed that Black Canadians were more likely to experience negative socioeconomic effects of the COVID-19 pandemic.^58^,^59^ These results speak to the need for fair distribution of COVID-19 preventive and treatment services.

Several studies^38^,^40^-^42^,^44^,^45^ in our scoping review described the low uptake of COVID-19 vaccines among Black Canadians. Vaccine hesitancy, recognized as a serious threat to public health, is significant among Black people. Some of the factors leading to vaccine hesitancy among Black Canadians are anti-Black racism in health care, distrust of the health care system and the failure to prioritize Black communities during vaccine rollouts.^40^,^42^ This is consistent with the findings of a systematic review from Canada^60^ and a meta-analysis from the USA.^61^ Statistics Canada reported that a much lower proportion of the Black population (56.4%) were very or somewhat willing to be vaccinated compared to White (77.7%) and South Asian (82.5%) populations.^62^ The distrust of COVID-19 vaccines is partly rooted in historical events of medical cruelty and unethical health research carried out on Black people, the perceived precipitous development of the vaccines, and community members’ lack of access to adequate information about the safety of the vaccines.^63^-^65^

A systematic review found that, given that many factors influence vaccine hesitancy, multicomponent interventions that incorporate intensified communication, culturally inclusive informational materials, community outreach and greater accessibility are the most reliable strategies to address this issue.^66^ Black people’s trust in the COVID-19 vaccine and its acceptance can be achieved by involving trusted community and faith leaders,^67^ providing culturally congruent materials and making vaccine information more accessible.^68^,^69^ In addition, a change in health policies and programs to garner trust and direct more attention to anti-Black racism will increase vaccine uptake in the Black Canadian community. Employing culturally representative health care personnel to inform Black people in the community can also influence acceptance of COVID-19 vaccine.^70^

The findings from this scoping review suggest that many Black Canadians had difficulties accessing COVID-19-related health care as a result of racism, systemic bias and socioeconomic vulnerabilities. Black Canadians largely perceive the health care system as racially and culturally alienating, and feel that the medical language and cultural barriers had a negative impact on their health care access;^62^ and this was exacerbated at the peak of the COVID-19 pandemic. The pandemic revealed the discrimination and racism that have long resulted in poor emotional, mental and physical health outcomes for African-Americans in the USA,^70^ with minority groups tending to receive lower standard of care than White people do, predisposing African-Americans to worse COVID-19 outcomes.^71^

Black Canadians’ relationships with health care personnel have been negatively affected by cultural differences, lack of cultural competence, dependence on the biomedical model and discrimination that has resulted in mistrust.^72^-^74^ Culturally sensitive interventions can enhance health care and patient outcomes,^75^,^76^ so it is critical to provide Black Canadians with a range of treatment options that incorporate culturally specific supports. Cultural awareness training for health care workers and employment of more Black health care workers would meaningfully contribute to overcoming cultural barriers to health care for Black people in Canada.

Our scoping review also found that Black Canadians and other minority groups encountered mental health distress during the pandemic.^38^,^47^,^50^,^51^ Existing discrepancies in mental health among Black people in Canada and African-Americans in the USA were exacerbated during the COVID-19 pandemic.^77^,^78^ Numerous factors, such as socioeconomic factors and access to mental health services, are responsible for the discrepancy.^78^ Resolving misinformation among Black Canadians through reliable sources and adapting tailored, multimodal and culturally intelligent messaging is important.

***Strengths and limitations***

To the best of our knowledge, this is the first scoping review that focusses on empirical research on the effects of the COVID-19 pandemic on Black people living in Canada. The small number of studies included (n = 17) demonstrates the lack of research on COVID-19 among Black people in Canada, suggesting the need for more studies.

## Conclusion

Our review revealed structural barriers, high rates of COVID-19 infections and low uptake of COVID-19 vaccines among Black Canadians, confirming research findings that the COVID-19 pandemic amplified health inequities, generated new barriers to health care, increased mistrust and reduced a sense of belonging among Black people.^8^,^17^,^79^ More research needs to be conducted to inform policies and programs to address the root causes of inequities. 

Some of the studies in this scoping review highlight the need to prioritize the equitable allocation of COVID-19 preventive measures and treatment. COVID-19 prevention strategies that are culturally appropriate and specific should also be made available and accessible. More generally, such initiatives should address the existing barriers associated with structural racism, medical distrust, educational inequities and health inequities. Canadian federal and provincial governments should implement strategies geared towards partnerships with Black-led and Black-focussed community groups to appropriately manage COVID-19 vaccine knowledge gaps and associated distrust factors and barriers, as sensitive and safe education implementation will increase vaccine confidence and herd immunity among Black communities to the benefit of society. Finally, collecting race-based data with the aim of resolving inequities and improving the health and well-being of Black people in Canada is essential to inform policies and address racial discrimination and access to health care services, as well as quality housing and employment. 

## Acknowledgements

The authors thank Mary Olukotun, PhD Student, Faculty of Nursing, University of Alberta, for her invaluable contributions in searching the databases for the reviewed articles.

## Funding

This work was funded by the Government of Canada, Department of Heritage Digital Citizenship Program (https://www.canada.ca/en/canadian-heritage/services/online -disinformation/digital-citizen-contribution-program.html).

## Conflicts of interest

The authors declare no competing interests.

## Authors’ contributions and statement

BS: Conceptualization, funding acquisition, supervision, writing – review & editing. 

AO: Data curation, formal analysis, writing – original draft. 

JK: Data curation, writing – review & editing. 

DA: Data analysis, writing – review & editing. 

MT: Review & editing.

AR: Review & editing.

AS: Review & editing.

DVM: Review & editing.

KO: Review & editing.

All authors read and agreed on the manuscript.

The content and views expressed in this article are those of the authors and do not necessarily reflect those of the Government of Canada.

## References

[B01] Pareek M, Bangash MN, Pareek N (2020). Ethnicity and COVID-19: an urgent public health research priority. Lancet.

[B02] Laurencin CT, McClinton A (2020). The COVID-19 pandemic: a call to action to identify and address racial and ethnic disparities. J Racial Ethn Health Disparities.

[B03] Millett GA, Jones AT, Benkeser D, et al (2020). Assessing differential impacts of COVID-19 on black communities. Ann Epidemiol.

[B04] Moore SE, Jones-Eversley SD, Tolliver WF, et al (2020). Six feet apart or six feet under: The impact of COVID-19 on the Black community. Death Stud.

[B05] Hooper M, poles AM, Stable EJ (2020). COVID-19 and racial/ethnic disparities. JAMA.

[B06] Coronavirus (COVID-19) related deaths by ethnic group, England and Wales [Internet]. Office for National Statistics.

[B07] Mensah J, Williams CJ (2022). Socio-structural injustice, racism, and the COVID-19 pandemic: a precarious entanglement among Black immigrants in Canada. Stud Soc Justice.

[B08] Risk for COVID-19 infection, hospitalization, and death by race/ethnicity [Internet]. National Center for Immunization and Respiratory Diseases (U.

[B09] Garg S, Kim L, Whitaker M, et al (2020). Hospitalization rates and characteristics of patients hospitalized with laboratory-confirmed coronavirus disease 2019 – COVID-NET, 14 states, March 1-30, 2020. Garg S, Kim L, Whitaker M, et al.

[B10] Yancy CW (2020). COVID-19 and African Americans. JAMA.

[B11] Khunti K, Platt L, Routen A, Abbasi K (2020). Covid-19 and ethnic minorities: an urgent agenda for overdue action. BMJ.

[B12] (2019). Racial discrimination in Britain, 1969–2017: a meta-analysis of field experiments on racial discrimination in the British labour market. Br J Sociol.

[B13] Gawthrop E The color of coronavirus: COVID-19 deaths by race and ethnicity in the U.S. S. [Internet]. Saint Paul (MN): APM Research Lab [Internet].

[B14] Picheta R Black people in the UK four times more likely to die from Covid-19 than white people, new data shows [Internet]. CNN.

[B15] Aldridge RW, Lewer D, Katikireddi SV, et al (2020). Black, Asian and minority ethnic groups in England are at increased risk of death from COVID-19: indirect standardisation of NHS mortality data. Wellcome Open Res.

[B16] Kolin DA, Kulm S, Christos PJ, Elemento O (2021). Clinical, regional, and genetic characteristics of Covid-19 patients from UK Biobank. PLos ONE.

[B17] Lo CH, Nguyen LH, Drew DA, et al (2021). Race, ethnicity, community-level socioeconomic factors, and risk of COVID-19 in the United States and the United Kingdom. EClinicalMedicine.

[B18] Daily status of COVID-19 cases by surveillance & epidemiology: Summary of COVID-19 counts in Toronto [Internet]. City of Toronto.

[B19] Census Profile, 2021 Census of population [Internet]. Statistics Canada.

[B20] Morency JD, Catalogue No Immigration and diversity: population projections for Canada and its regions, 2011 to 2036 [modified 2022 Jan 11; cited 2023 Feb 09]. Immigration and diversity: population projections for Canada and its regions, 2011 to 2036 [modified 2022 Jan 11; cited 2023 Feb 09]. [Statistics Canada Catalogue No.

[B21] Robertson A, Prescod C, Brooks D, et al Statement from Black health leaders on COVID-19’s impact on Black communities in Ontario [Internet]. Alliance for Healthier Communities.

[B22] Tai DB, Shah A, Doubeni CA, Sia IG, Wieland ML (2021). The disproportionate impact of COVID-19 on racial and ethnic minorities in the United States. Clin Infect Dis.

[B23] Wallis C Why racism, not race is a risk factor for dying of COVID-19 [Internet]. Scientific American.

[B24] Njoku A, Joseph M, Felix R (2021). Changing the narrative: structural barriers and racial and ethnic inequities in COVID-19 vaccination. Int J Environ Res Public Health.

[B25] Jr L, Enwere M, Williams J, et al (2020). Black–White risk differentials in COVID-19 (SARS-COV2) transmission, mortality and case fatality in the United States: translational epidemiologic perspective and challenges. Int J Environ Res Public Health.

[B26] Hou F, Frank F, Schimmele C Economic impact of COVID-19 among visible minority groups [Internet]. Statistics Canada.

[B27] Chang S, Pierson E, Koh PW, et al (2021). Mobility network models of COVID-19 explain inequities and inform reopening. Nature.

[B28] McCormack GR, Doyle-Baker PK, Petersen JA, Ghoneim D (2020). Parent anxiety and perceptions of their child’s physical activity and sedentary behaviour during the COVID-19 pandemic in Canada. Prev Med Rep.

[B29] (2020). Sociodemographic determinants of occupational risks of exposure to COVID-19 in Canada. Can Rev Sociol.

[B30] Reyes M (2020). The disproportional impact of COVID-19 on African Americans. Health Hum Rights.

[B31] Arksey H, O’Malley L (2005). Scoping studies: towards a methodological framework. Int J Soc Res Methodol.

[B32] Tricco AC, Lillie E, Zarin W, et al PRISMA extension for scoping reviews (PRISMA-ScR): checklist and explanation. Ann Intern Med.

[B33] Page MJ, McKenzie JE, Bossuyt PM, et al (2021). et al. BMJ.

[B34] Braun V, Clarke V (2014). What can “thematic analysis” offer health and wellbeing researchers. Braun V, Clarke V.

[B35] Ahmed R, Jamal O, Ishak W, Nabi K, Mustafa N (2021). Racial equity in the fight against COVID-19: a qualitative study examining the importance of collecting race-based data in the Canadian context. Trop Dis Travel Med Vaccines.

[B36] Pongou R, Ahinkorah BO, Mabeu MC, Agarwal A, Maltais S, Yaya S (2022). Examining the association between reported COVID-19 symptoms and testing for COVID-19 in Canada: a cross-sectional survey. BMJ Open.

[B37] Rocha R, Shringler B, Montpetit J Montreal’s poorest and most racially diverse neighbourhoods hit hardest by COVID-19, data analysis shows: Census data shows how race, housing and income correlate to spread of COVID-19. CBC News.

[B38] Kemei J, Tulli M, Olanlesi-Aliu A, Tunde-Byass M, Salami B (2023). Impact of the COVID-19 pandemic on Black communities in Canada. Int J Environ Res Public Health.

[B39] Choi KH, Denice P, Haan M, Zajacova A (2021). Studying the social determinants of COVID-19 in a data vacuum. Can Rev Sociol.

[B40] Kemei J, Alaazi DA, Olanlesi-Aliu A, et al (2023). What contributes to COVID-19 online disinformation among Black Canadians: a qualitative study. CMAJ Open.

[B41] Richard L, Liu M, Jenkinson JI, et al COVID-19 vaccine coverage and sociodemographic, behavioural and housing factors associated with vaccination among people experiencing homelessness in Toronto, Canada: a cross-sectional study. Richard L, Liu M, Jenkinson JI, et al.

[B42] Gerretsen P, Kim J, Quilty L, et al (2021). Vaccine hesitancy is a barrier to achieving equitable herd immunity among racial minorities. Vaccine hesitancy is a barrier to achieving equitable herd immunity among racial minorities. Front Med (Lausanne).

[B43] COVID-19 vaccine confidence: Black Canadian perspectives [Internet]. Innovative Research Group.

[B44] nat JM, Farahi SM, Bakombo SM, et al (2023). Vaccine mistrust among Black individuals in Canada: the major role of health literacy, conspiracy theories, and racial discrimination in the healthcare system. J Med Virol.

[B45] Etowa J, Ghose B, Etowa E, Dabone C (2023). COVID-19 vaccine willingness among African, Caribbean, and Black People in Ottawa, Ontario. COVID.

[B46] Lei L, Guo S (2022). Beyond multiculturalism: revisioning a model of pandemic anti-racism education in post-Covid-19 Canada. Int J Anthropol Ethnol.

[B47] Miconi D, Li ZY, et al (2020). Ethno-cultural disparities in mental health during the COVID-19 pandemic: a cross-sectional study on the impact of exposure to the virus and COVID-19-related discrimination and stigma on mental health across ethno-cultural groups in Quebec (Canada). BJPsych Open.

[B48] Etowa J, Abrha G, Etowa E, Ghose B (2021). Healthcare during COVID-19 in Canada: need for strengthening providers’ capacity for best practices in African, Caribbean and Black community service provision. Am J Public Health Res.

[B49] Noble A, Owens B, Thulien N, Suleiman A (2022). “I feel like I'm in a revolving door, and COVID has made it spin a lot faster”: The impact of the COVID-19 pandemic on youth experiencing homelessness in Toronto, Canada. PLoS One.

[B50] Pang Y, Li M, Robbs C, et al (2021). Risk factors for mental health symptoms during the COVID-19 pandemic in ophthalmic personnel and students in USA (& Canada): a cross-sectional survey study. BMC Psychiatry.

[B51] Pongou R, Ahinkorah BO, Maltais S, Mabeu MC, Agarwal A, Yaya S (2022). Psychological distress during the COVID-19 pandemic in Canada. PLoS One.

[B52] Gupta S, Aitken N COVID-19 mortality among racialized populations in Canada and its association with income [Internet]. Statistics Canada.

[B53] Williamson EJ, Walker AJ, Bhaskaran K, et al (2020). Factors associated with COVID-19-related death using OpenSAFELY. Nature.

[B54] Rubin-Miller L, Alban C, Artiga S, Sullivan S COVID-19 racial disparities in testing, infection, hospitalization, and death: analysis of epic patient data. Kaiser Family Foundation.

[B55] Lieberman-Cribbin W, Tuminello S, Flores RM, Taioli E (2020). Disparities in COVID-19 testing and positivity in New York City. Am J Prev Med.

[B56] COVID 19: ethno-racial identity & income [Internet]. City of Toronto.

[B57] Report: COVID-19 and racial identity in Ottawa. Ottawa Public Health.

[B58] Impact of COVID-19: Black Canadian perspectives [Internet]. African-Canadian Civic Engagement Council (ACCEC); Innovative Research Group.

[B59] Adrien A, Springmann V Ingaux face la pandmie: populations racises et la COVID-19 [Internet]. Direction rgionale de sant publique de Montral.

[B60] nat JM, Noorishad PG, Bakombo SM, et al (2022). A systematic review on vaccine hesitancy in Black communities in Canada: critical issues and research failures. Vaccines (Basel).

[B61] Dhanani LY, Franz B (2022). A meta-analysis of COVID-19 vaccine attitudes and demographic characteristics in the United States. Public Health.

[B62] COVID-19 vaccine willingness among Canadian population groups [Internet]. Statistics Canada.

[B63] “We were out ahead of public health”: Leading COVID-19 vaccine equity for Black communities across Canada. NCCDH; St. Francis Xavier University.

[B64] Crawshaw J, Konnyu K, Castillo G, et al Factors affecting COVID-19 vaccination acceptance and uptake among the general public: a living behavioural science evidence synthesis (v5, Aug 31st, 2021) [Internet]. Ottawa Hospital Research Institute.

[B65] Bertin P, Nera K, e S (2020). Conspiracy beliefs, rejection of vaccination, and support for hydroxychloroquine: a conceptual replication-extension in the COVID-19 pandemic context. Front Psychol.

[B66] Adeagbo M, Olukotun M, Musa S, et al (2022). Improving COVID-19 vaccine uptake among Black populations: a systematic review of strategies. Int J Environ Res Public Health.

[B67] Abdul-Mutakabbir JC, Casey S, Jews V, et al (2021). A three-tiered approach to address barriers to COVID-19 vaccine delivery in the Black community. Lancet Glob Health.

[B68] A guide for community partners: increasing COVID-19 vaccine uptake among racial and ethnic minority communities [Internet]. U.S. Department of Health and Human Services/CDC National Center for Immunization and Respiratory Diseases.

[B69] Feifer RA, Bethea L, White EM (2021). Racial disparities in COVID-19 vaccine acceptance: building trust to protect nursing home staff and residents. J Am Med Dir Assoc.

[B70] Dada D, Djiometio JN, McFadden SM, et al (2022). Strategies that promote equity in COVID-19 vaccine uptake for Black communities: a review. J Urban Health.

[B71] Laurencin CT, Walker JM (2020). A pandemic on a pandemic: racism and COVID-19 in Blacks. Cell Syst.

[B72] Martin D, Miller AP, e A, Caron NR, e B, Marchildon GP (2018). Canada’s universal health-care system: achieving its potential. Lancet.

[B73] Crooks VA, Schuurman N (2012). Interpreting the results of a modified gravity model: examining access to primary health care physicians in five Canadian provinces and territories. BMC Health Serv Res.

[B74] Vines B Addressing COVID-19 vaccine hesitancy among Black Americans [Internet]. Vines B.

[B75] Hardeman RR, Medina EM, Kozhimannil KB (2016). Structural racism and supporting Black lives—the role of health professionals. N Engl J Med.

[B76] Armstrong K, Putt M, Halbert CH, et al (2013). Prior experiences of racial discrimination and racial differences in health care system distrust. Med Care.

[B77] Snowden LR, Snowden JM (2021). Coronavirus trauma and African Americans’ mental health: seizing opportunities for transformational change. Int J Environ Res Public Health.

[B78] Thomeer MB, Moody MD, Yahirun J (2023). Racial and ethnic disparities in mental health and mental health care during the COVID-19 pandemic. J Racial Ethn Health Disparities.

[B79] Reyes M (2020). The disproportional impact of COVID-19 on African Americans. Health Hum Rights.

